# Supplementary material for: Adenine base editing-mediated exon skipping restores dystrophin in humanized Duchenne mouse model
Source: Nat Commun. 2024 Jul 15;15:5927. doi: 10.1038/s41467-024-50340-x (PMC11251194; doi:10.1038/s41467-024-50340-x)
Supplement: Supplementary file 1 — Supplementary Information [file 41467_2024_50340_MOESM1_ESM.pdf]

Supplementary Information

Title: Adenine base editing-mediated exon skipping restores dystrophin in humanized Duchenne mouse model

Authors:

Jiajia Lin[1†], Ming Jin[1†], Dong Yang[2†], Zhifang Li[3†], Qingquan Xiao[2], Yin Wang[2], Yuyang Yu[2], Yu Zhang[2], Xiumei Zhang[2], Zhurui Shao[2], Linyu Shi[2], Shu Zhang[5], Wanjin Chen[1], Ning Wang[1*], Shiwen Wu[5*], Hui Yang[2,4*], Chunlong Xu[3,4*], Guoling Li[1, 2*]

†These authors contributed equally to this work.

Affiliations:

1. Department of Neurology, First Affiliated Hospital, Fujian Medical University, Fuzhou, China.

2. HuidaGene Therapeutics Inc., Shanghai, China.

3. Lingang Laboratory, Shanghai, China.

4. Shanghai Center for Brain Science and Brain-Inspired Technology, Shanghai, China.

5. Department of Neurology, First Medical Center of Chinese PLA General Hospital, Beijing, China.

†These authors contributed equally to this work.

*Correspondences: guolingli@huidagene.com(G.L.), xucl@lglab.ac.cn(C.X.), huiyang@huidagene.com(H.Y.), wu_shiwen@outlook.com(S.W.), ningwang@fjmu.edu.cn (N.W.)

This PDF file includes:

Supplementary Figure 1 to 18

Fig. S1: The muscle pathology and motor function of DMD$^{\Delta mE5051,KIhE50/Y}$ mice at different ages.

Fig. S2: ABE-mediated A-to-G editing in other exon splice sites of *DMD* gene.

Fig. S3: Comparison of the editing efficiencies of different ABE systems split with various intein sequences *in vitro*.

Fig. S4: Off-target analysis in HEK293T.

Fig. S5: Deep-seq read analysis for ABE1 and ABE2-edited splice sites of human *DMD* exon 50.

Fig. S6: Rescue of dystrophin expression following intramuscular (IM) injection of ABE systems after 6 weeks.

Fig. S7: Analysis of dystrophin protein level and Dys+ fibers after intraperitoneally delivery of AAV-ABE2 in DMD mice.

Fig. S8: Immunostaining of dystrophin in heart, TA, and DI tissues 6 weeks after IP injection of AAV-ABE2.

Fig. S9: Toxicity response to AAV-ABE treatment after IP injection.

Fig. S10: Base editing analysis of satellite cells from ABE2-treated DMD mice.

Fig. S11: Gel electrophoresis analysis of RNA exon skipping events after 6-week and 10-month ABE2 treatment in DMD mice.

Fig. S12: Analysis of dystrophin protein level and Dys+ fibers after intravenously delivery in DMD mice.

Fig. S13: Immunostaining of dystrophin in heart, TA, and DI tissues 6 weeks and 10 months after IV injection of AAV-ABE2.

Figure S14. Histological analysis after systemic delivery of ABE2 in DMD mice.

Fig. S15. Toxicity response to AAV-ABE2 treatment after IV injection.

Fig. S16. Echocardiography was used to assess the cardiac function of DMD mice after systemic delivery of ABE2.

Fig. S17. Flow cytometry gating strategy.

Fig. S18. Uncropped images.

Supplementary Table 1 to 2

Table S1: Primer sequence.

Table S2: Target sgRNA sequences.

Supplementary Note 1

Supplementary Note 1. Sequences of ABE1 and ABE2.

Supplementary Figures

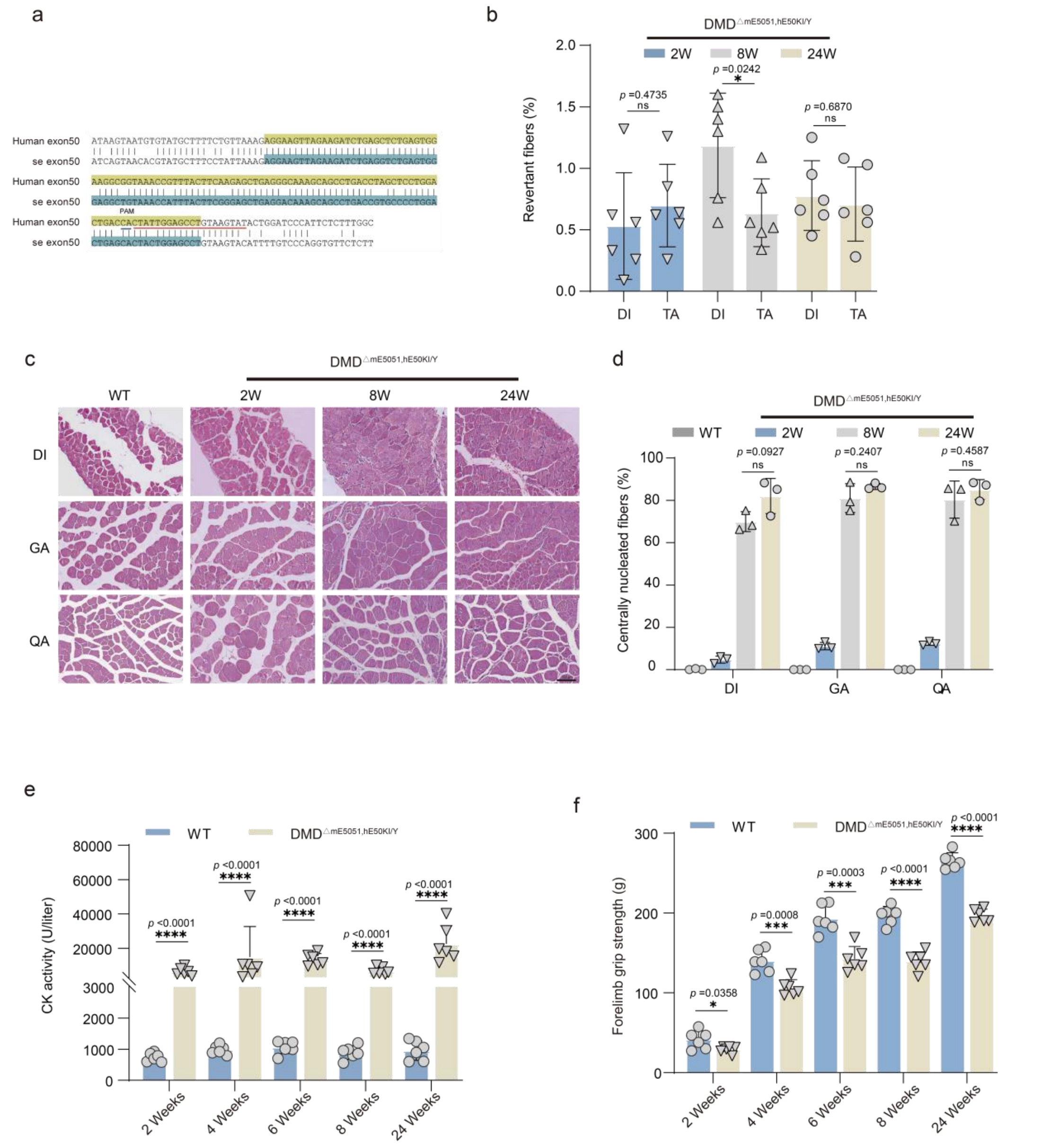

Fig. S1: The muscle pathology and motor function of DMD$^{\Delta mE5051,\ KIhE50/Y}$ mice at different ages.

a, Alignment of human and mouse exon 50 sequence. Human exon 50 (Yellow labeling), mouse exon 50 (Blue label), sgRNA (magenta line), Protospacer adjacent motif (Red line). **b,** Statistical analysis of the number of revertant fibers. **c,** H&E staining of diaphragm (DI), gastrocnemius (GA), and quadriceps (QA) muscle of WT and different age of DMD mice. Wild-type (WT) mice as control. Scale bar,100 μm. **d,** Statistical analysis of nuclear migration in H&E staining. n=3 independent biological replicates. **e,** Serum creatine kinase (CK), a marker

of muscle damage and membrane leakage, was measured in WT and DMD mice at the ages from 2 weeks to 24 weeks. n=6 independent biological replicates. **f,** The forelimb grip strength testing to measure muscle performance of WT and DMD mice at the ages from 2 weeks to 24 weeks. n=6 independent biological replicates. Data are presented as mean ± s.d. Each dot represents an individual mouse. Significance is indicated by asterisk and determined using unpaired two-tailed Student's t test. * $P < 0.05$. *** $P < 0.001$. **** $P < 0.0001$, Ns represents not statistically significant. Source data are provided as a Source Data file.

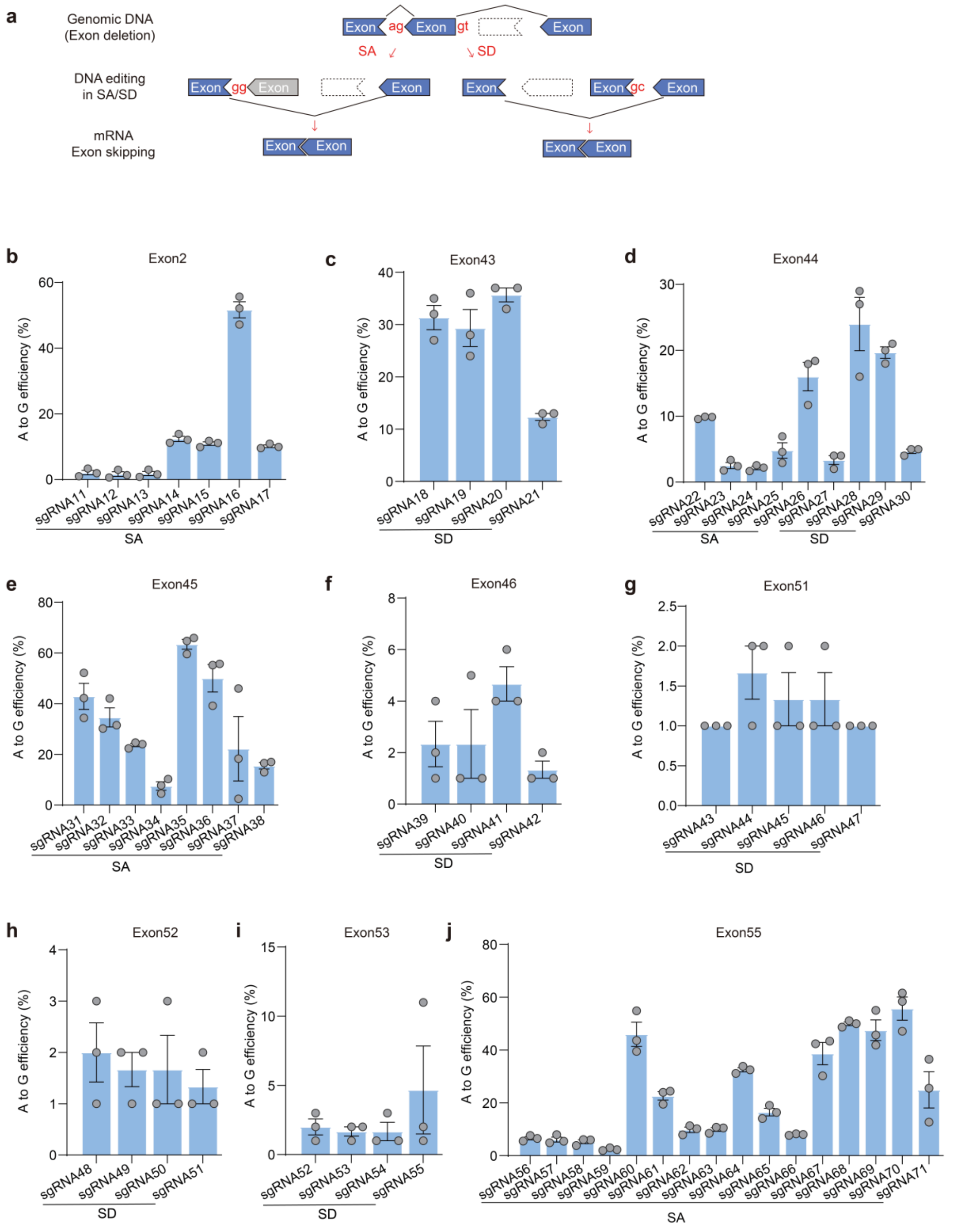

Fig. S2: ABE-mediated A-to-G editing in other exon splice sites of *DMD* gene.

a, Schematic diagram of nucleotide editing strategy in different exon splice sites. Deletion of exon in the *Dmd* gene generates a premature stop codon in next exon. Restoration of the correct open reading frame (ORF) can be obtained by skipping of exon splice donor (SD) or splice acceptor (SA). Percentages of DNA editing in *DMD* "hotspot" exon in HEK293T, including exon 2 (**b**), exon 43 (**c**), exon 44 (**d**), exon 45 (**e**), exon 46 (**f**), exon 51 (**g**), exon 52 (**h**), exon 53 (**i**) and

exon 55 (**j**). Data are presented as mean ± s.d (n=3 independent biological replicates). Source data are provided as a Source Data file.

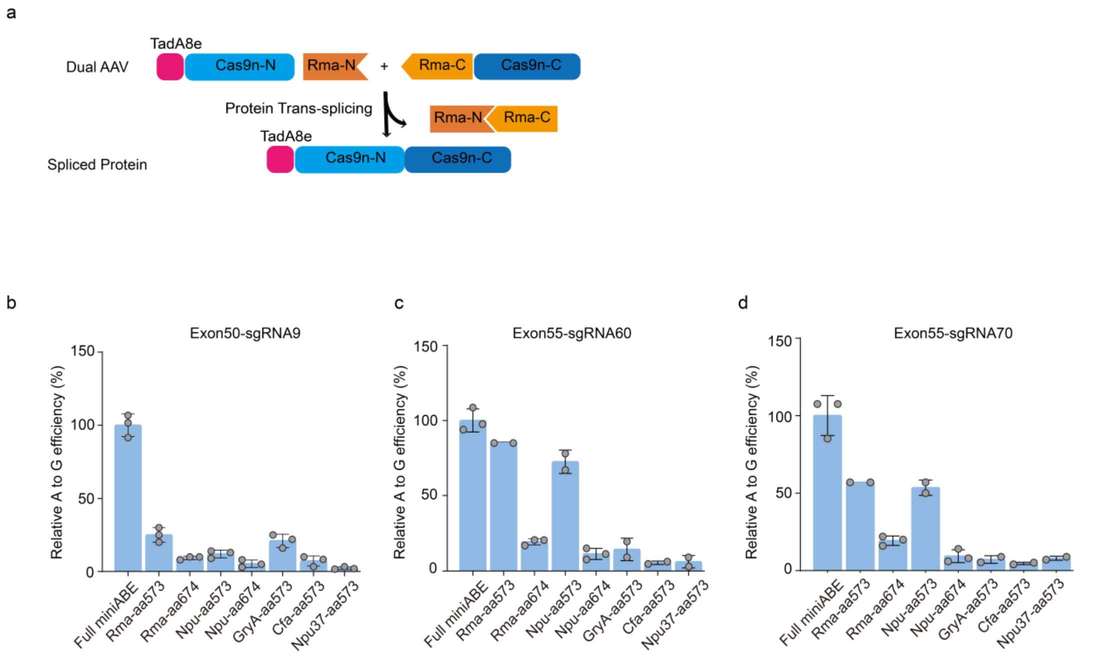

Fig. S3: Comparison of the editing efficiencies of different ABE systems split with various intein sequences in vitro.

a, Intein reconstitution strategy. Dual adeno-associated virus (AAV) vectors separately encoded protein fragments fused to split intein halves splice to reconstitute full-length protein following co-expression; Comparison of the editing efficiencies in *DMD* gene exon 50 (**b**), and exon 55 (**c** and **d**) with split ABE via various intein. Data are normalized with full-length protein and presented as mean ± s.d (n=2 or 3 independent biological replicates). Source data are provided as a Source Data file.

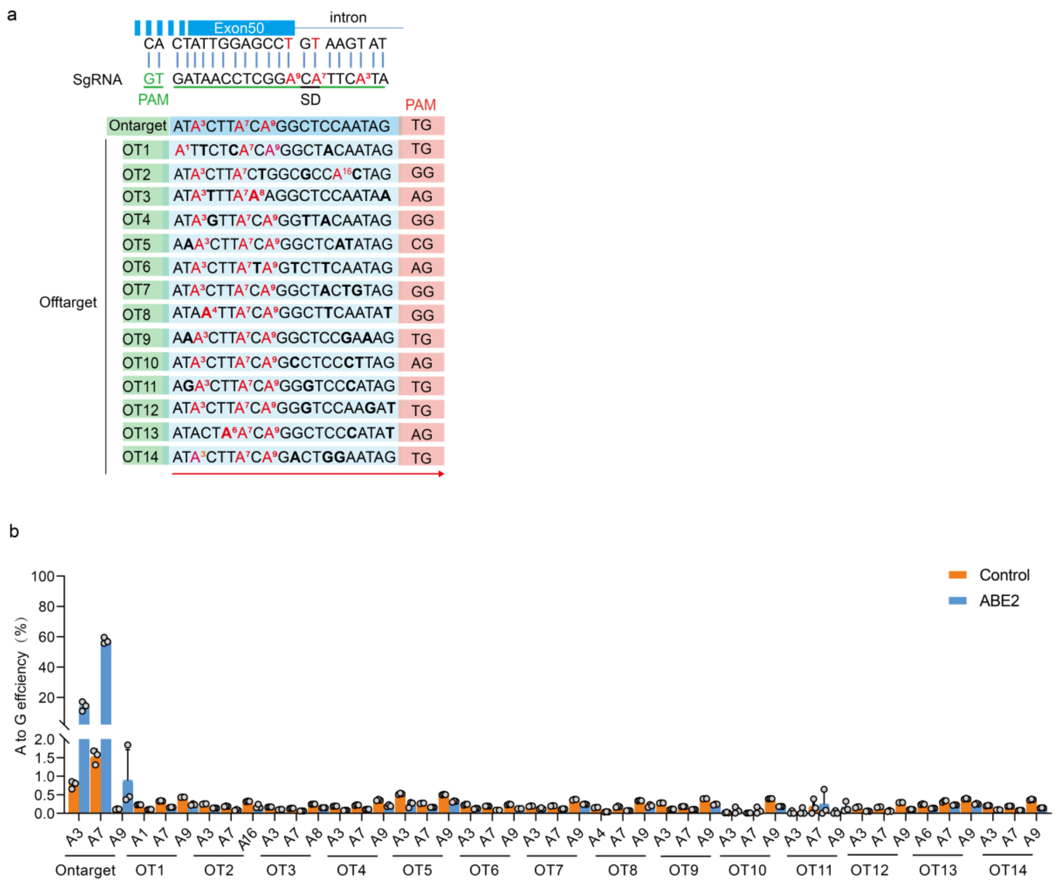

Fig. S4: Off-target analysis in HEK293T.

a, Alignment of the top 14 off-target sites in human genomic DNA. Potential off-target adenines are highlighted in red font, while black highlighted characters represent bases that are mismatched with on-target gRNA; **b,** Percentages of adenine editing in the all 14 potential off-target sites. Data are presented as mean ± s.d (n=3 independent biological replicates). Source data are provided as a Source Data file.

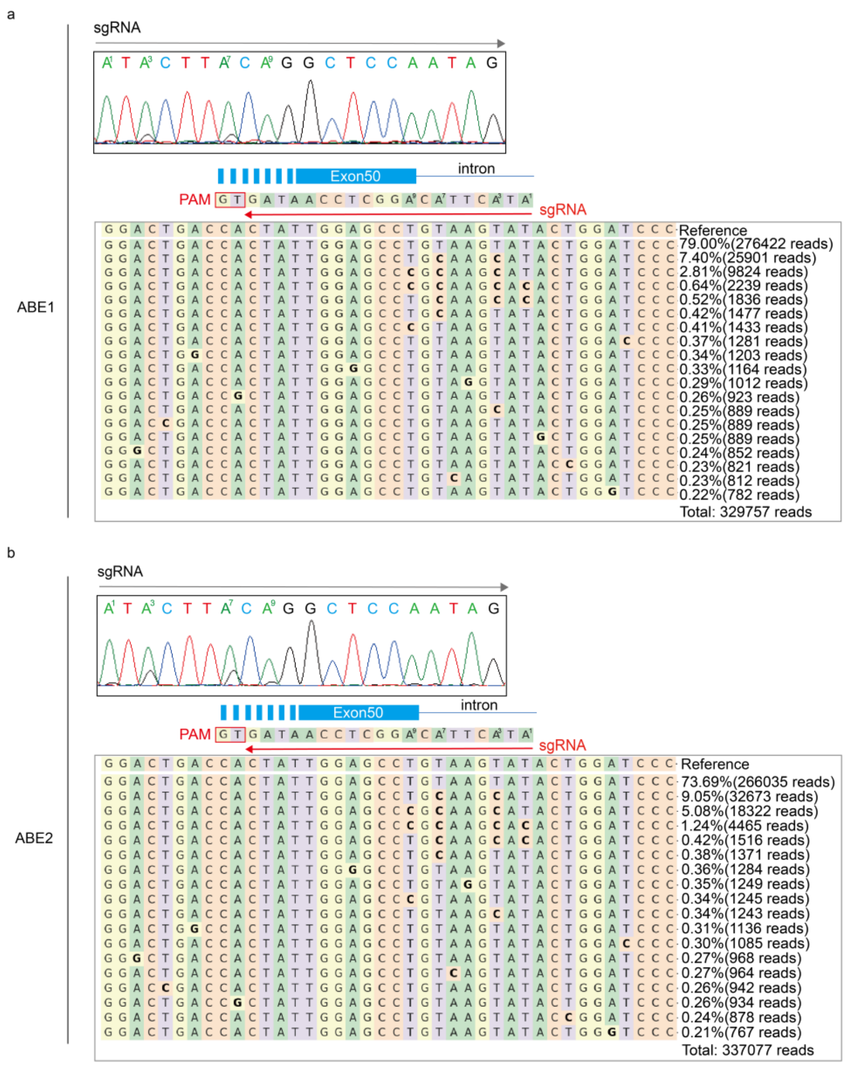

Fig. S5: Deep-seq read analysis for ABE1 and ABE2-edited splice sites of human *DMD* exon 50.

Chromatogram and deep-seq reads results for ABE1- (**a**) and ABE2-edited (**b**) splice sites of human *DMD* exon 50, respectively. The red arrow indicates the direction of the sgRNA, and the red box represents the PAM recognition sequence.

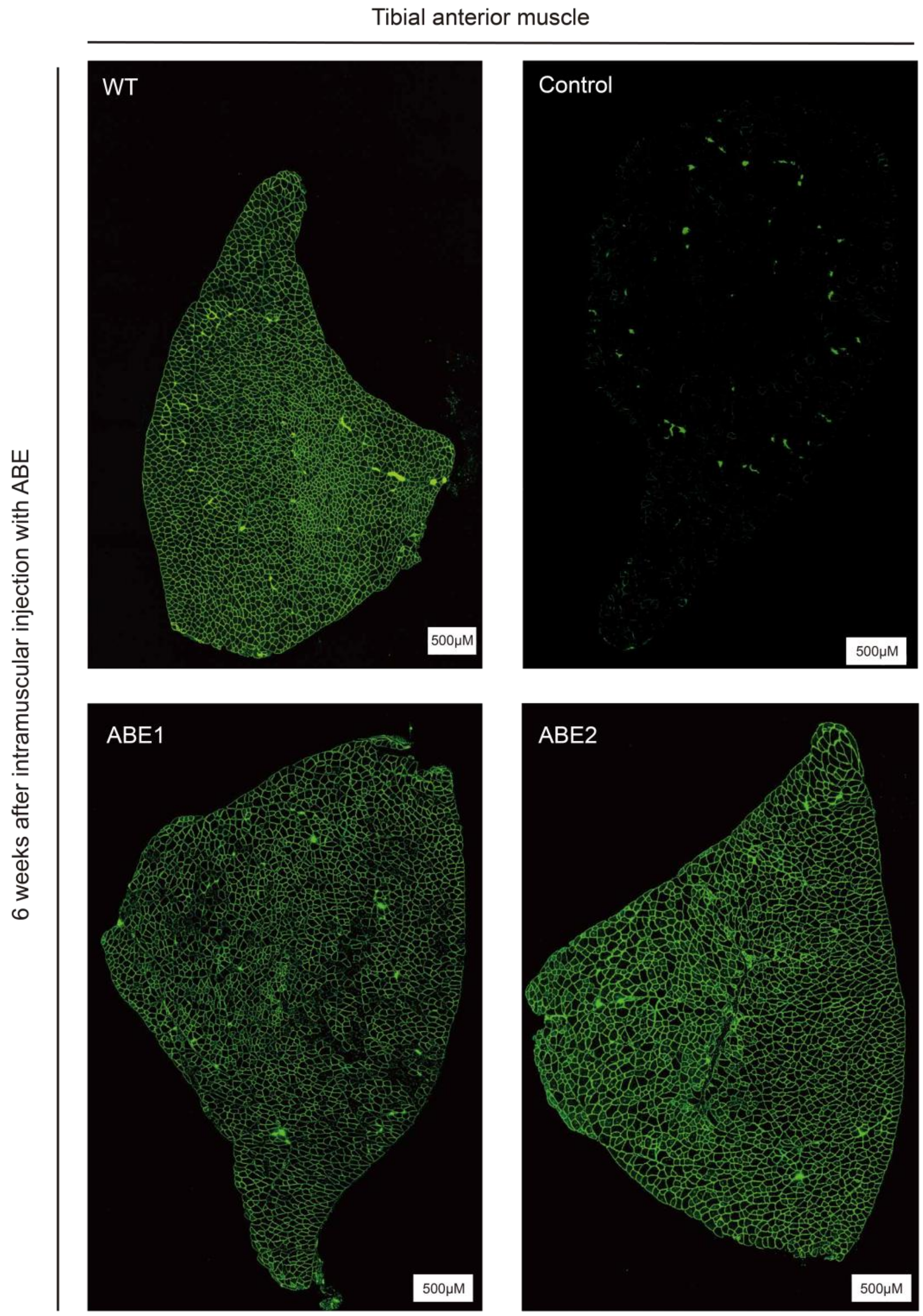

Fig. S6: Rescue of dystrophin expression following intramuscular (IM) injection of ABE systems after 6 weeks.

Dystrophin immunohistochemistry of entire tibialis anterior (TA) muscle. Control mice were injected with saline. Dystrophin is shown in green. Scale bar, 500 μm.

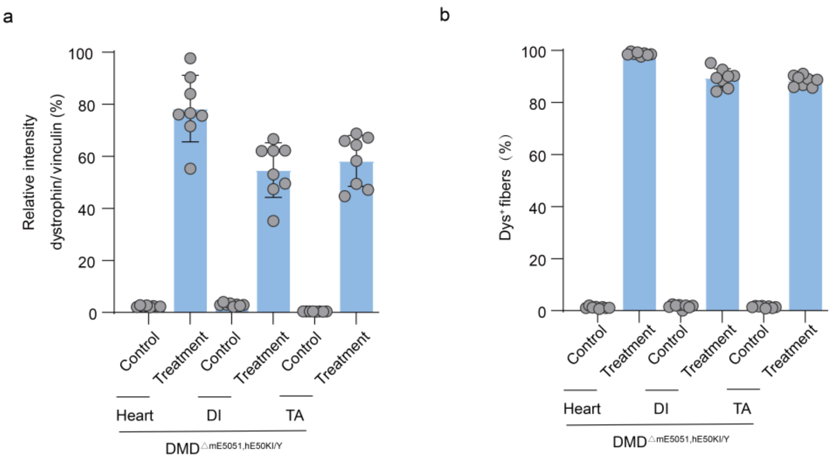

Fig. S7: Analysis of dystrophin protein level and Dys+ fibers after intraperitoneally delivery of AAV-ABE2 in DMD mice.

a, Relative dystrophin intensity was calibrated against the internal vinculin control and normalized to the wild-type dystrophin level. **b,** Percentage of Dys+ tissue area in tibialis anterior (TA) from treated and untreated DMD$^{\Delta mE5051,KIhE50/Y}$ mice. Data are presented as mean ± s.d (n=8 independent biological replicates). Significance is indicated with asterisk and determined using unpaired two-tailed Student's t test. Ns, not statistically significant. Source data are provided as a Source Data file.

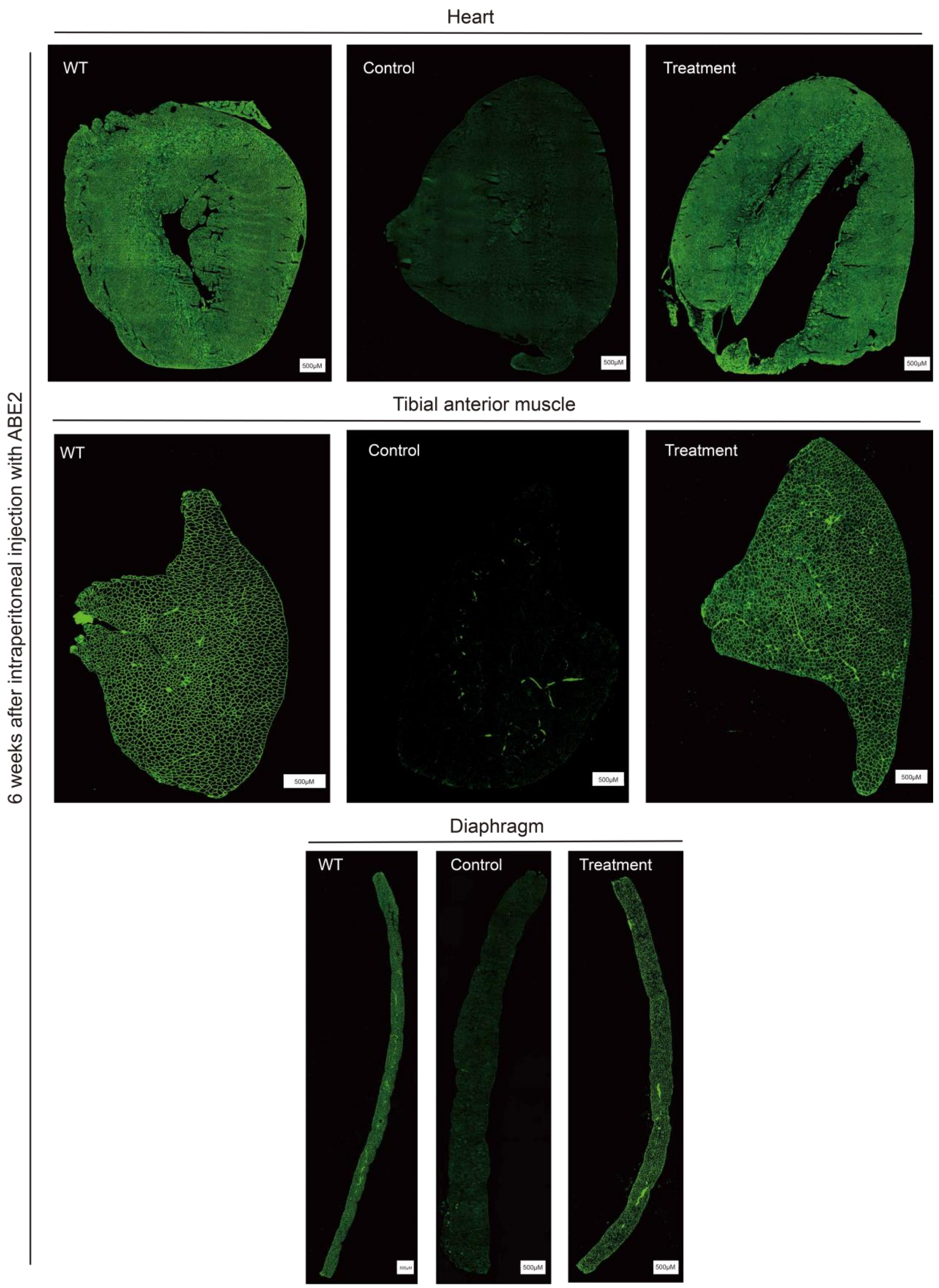

Fig.S8

Fig. S8: Immunostaining of dystrophin in heart, TA, and DI tissues 6 weeks after IP injection of AAV-ABE2.

Whole-muscle scanning of diaphragm (DI), tibialis anterior (TA) and and heart of DMD$^{\Delta mE5051,KIhE50/Y}$ mice 6 weeks after systemic delivery of ABE2 particles.

Control mice were injected with saline. Dystrophin is shown in green. Scale bar, 500 µm. Images shown in both Fig. 4f and Fig. S7 were obtained from the same tissue at 20× magnification. Fig. 4f showed the local region staining image rather than the reconstituted whole-tissue scanning image in Fig. S8.

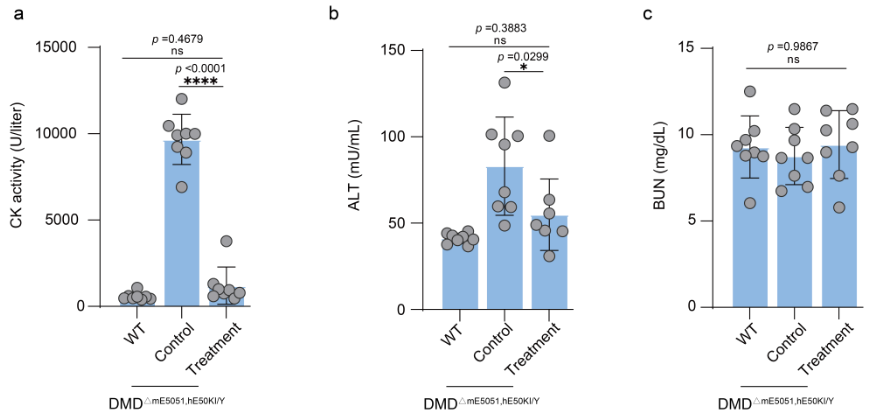

Fig. S9: Toxicity response to AAV-ABE treatment after IP injection.
Characterization of creatine kinase (CK) (**a**), alanine aminotransferase (ALT) (**b**) and blood urea nitrogen (BUN) (**c**) activity after intraperitoneal injection of AAV-ABE2 (n=8 independent biological replicates). Data are shown as mean ± s.d. Significance is indicated by asterisk and determined using unpaired two-tailed Student's t test. * $P < 0.05$. **** $P < 0.0001$, Ns represents not statistically significant. Source data are provided as a Source Data file.

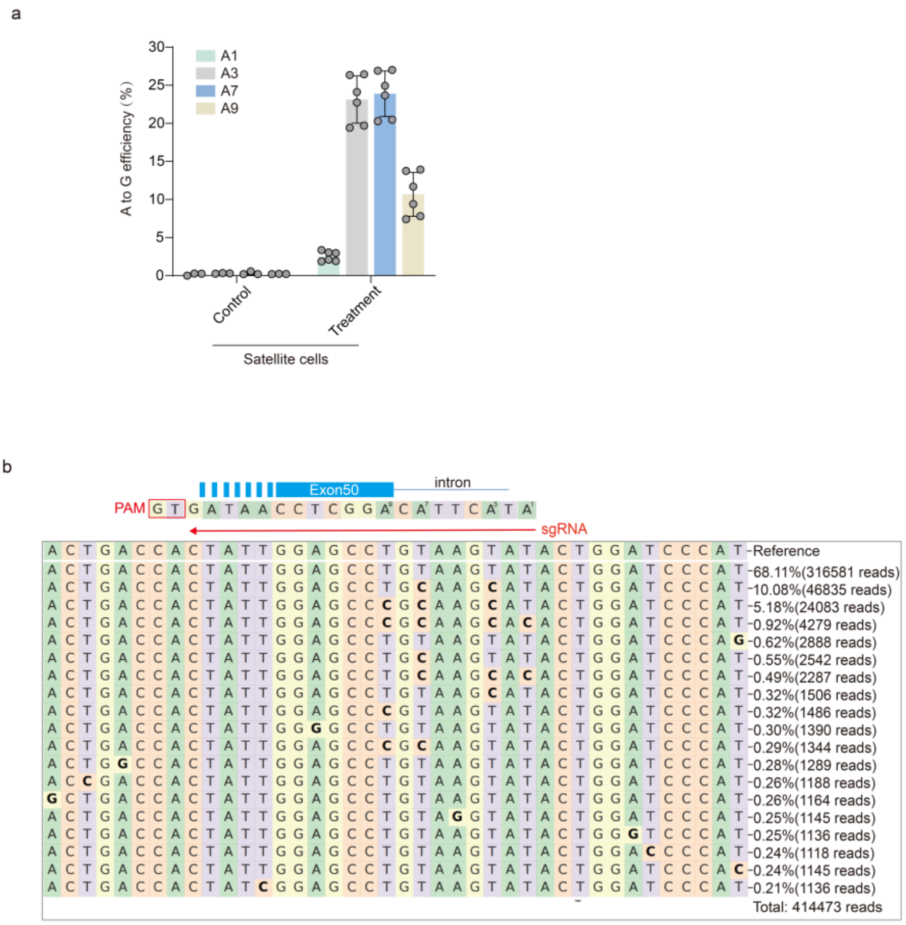

Fig. S10: Base editing analysis of satellite cells from ABE2-treated DMD mice.

a, A-to-G conversion efficiency for adenine on the target splice site of human *DMD* exon 50. **b,** Deep-seq read analysis of ABE2-edited satellite cells. Source data are provided as a Source Data file.

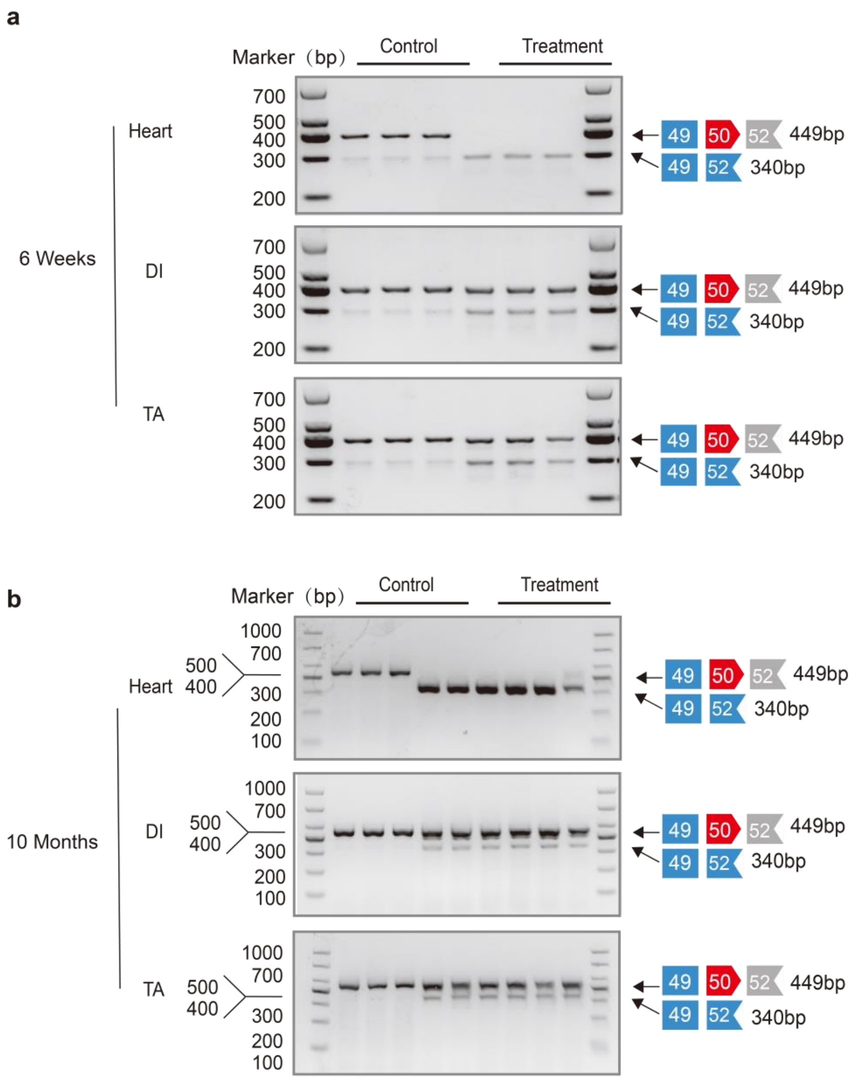

Fig. S11: Gel electrophoresis analysis of RNA exon skipping events after 6-week and 10-month ABE2 treatment in DMD mice.

Gel electrophoresis results for *DMD* exon 50 skipping induction by ABE2 at 6-week (**a**) and 10-month (**b**) post AAV injection in DMD$^{\Delta mE5051,KIhE50/Y}$ mice. Source data are provided as a Source Data file.

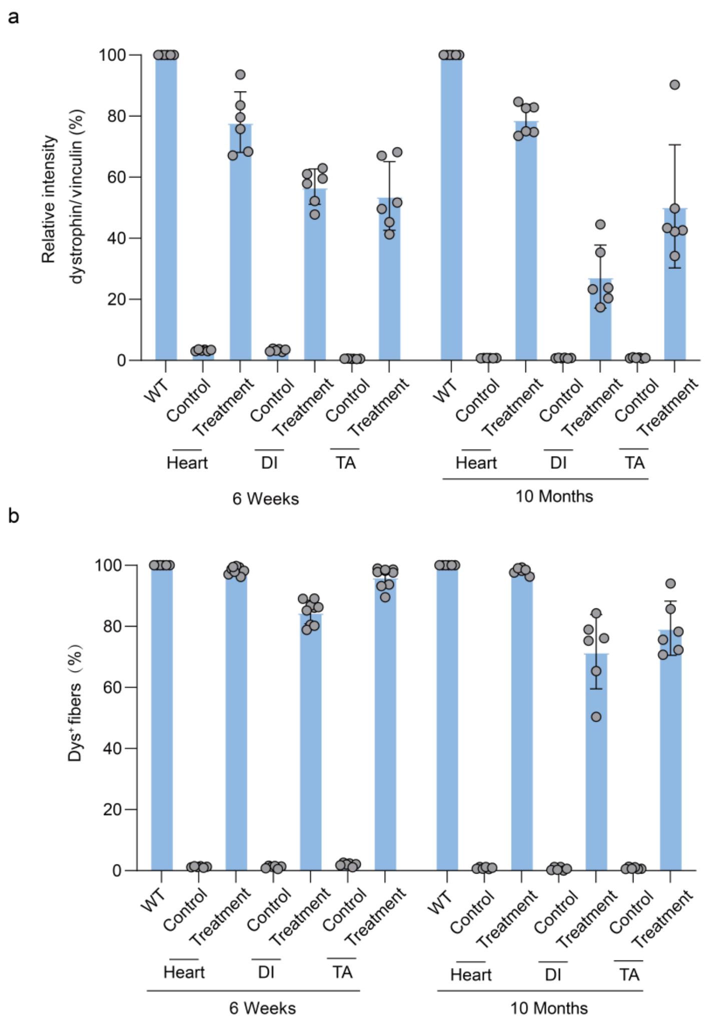

Fig. S12: Analysis of dystrophin protein level and Dys+ fibers after intravenously delivery in DMD mice.

a, Relative dystrophin intensity was calibrated against the internal vinculin control and normalized to the wildtype dystrophin level. **b,** Percentage of Dys+ tissue area in TA, DI and heart from treated and untreated DMD$^{\Delta mE5051,KIhE50/Y}$ mice. Data are shown as mean ± s.d (n=6 independent biological replicates for 10-month post-treatment group and n=9 independent biological replicates for 6-week post-treatment group). Significance is indicated by asterisk and determined using unpaired two-tailed Student’s t test. Ns represents not statistically significant. Source data are provided as a Source Data file.

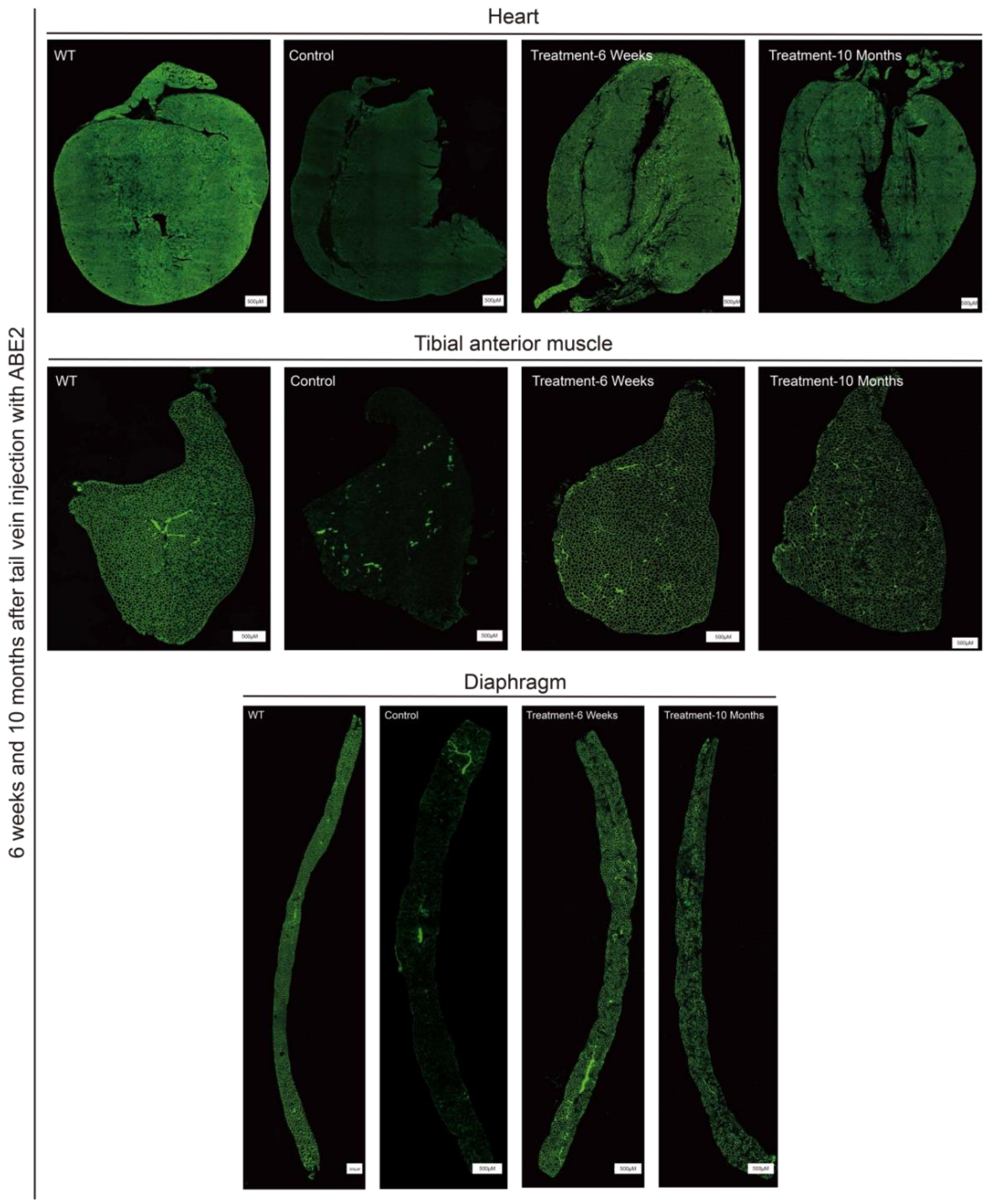

Fig. S13: Immunostaining of dystrophin in heart, TA, and DI tissues 6 weeks and 10 months after IV injection of AAV-ABE2.

Whole-muscle scanning of tibialis anterior (TA), diaphragm (DI), and heart muscle of $DMD^{\Delta mE5051,KIhE50/Y}$ mice 6 weeks and 10 months after systemic delivery of ABE2 particles. Control DMD mice were injected with saline. Dystrophin is shown in green. Scale bar, 500 μm. Images shown in both Fig. 5e and Fig. S13 were obtained from the same tissue at 20× magnification. Fig. 5e showed the local region staining image rather than the reconstituted whole-tissue scanning image in Fig. S13.

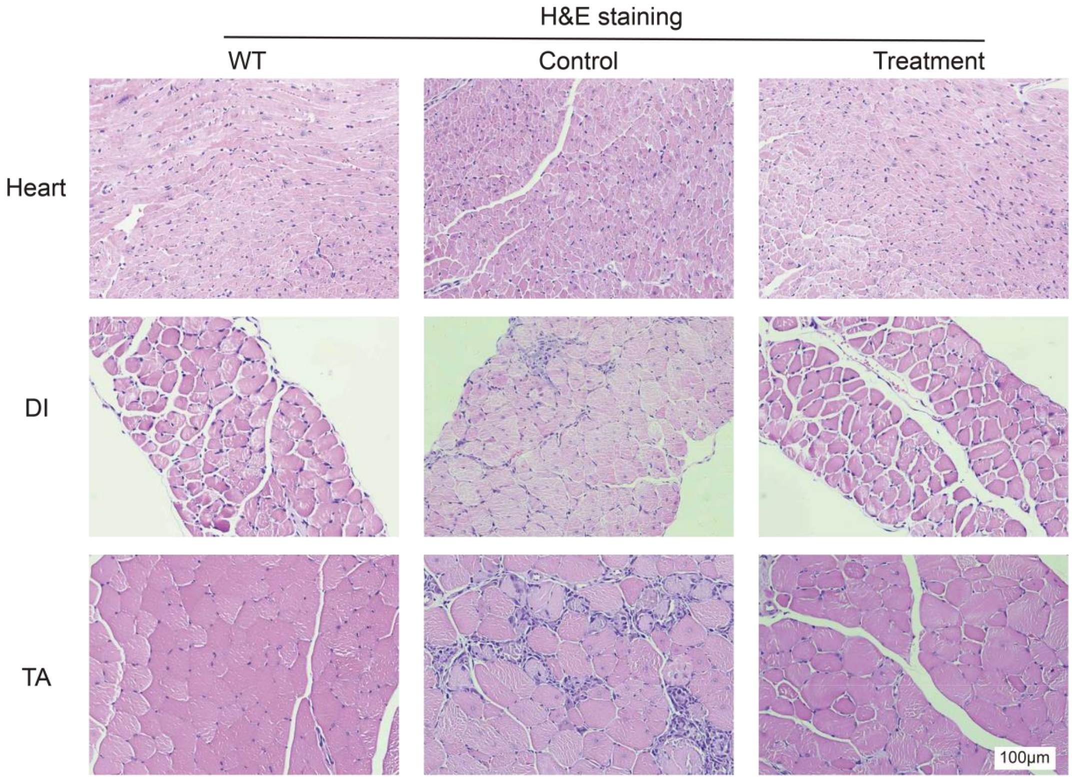

Figure S14. Histological analysis after systemic delivery of ABE2 in DMD mice.

H&E staining of tibialis anterior (TA), diaphragm (DI), and heart of wild-type (WT), untreated and ABE2-treated DMD$^{\Delta mE5051,KIhE50/Y}$ mice at 6-week after intravenous injection. Scale bars, 100 μm.

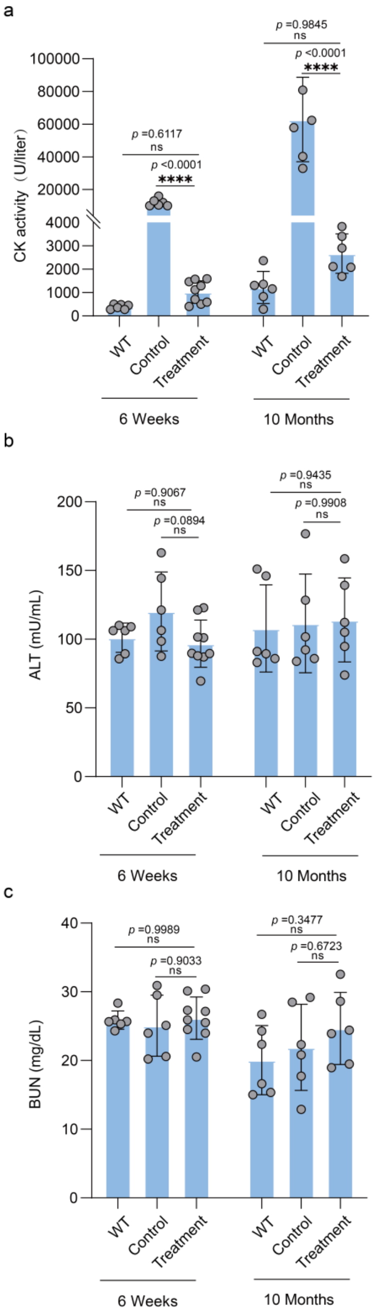

Fig. S15: Toxicity response to AAV-ABE2 treatment after IV injection.

CK (**a**), ALT (**b**) and BUN (**c**)activity was detected after intraperitoneal injection with ABE2. Data are presented as mean ± s.d (n=6 independent biological replicates for 10-month post-treatment group and n=9 independent biological replicates for 6-week post-treatment group). Significance is indicated by asterisk and determined using unpaired two-tailed Student's t test. **** P < 0.0001, Ns represents not statistically significant. Source data are provided as a Source Data file.

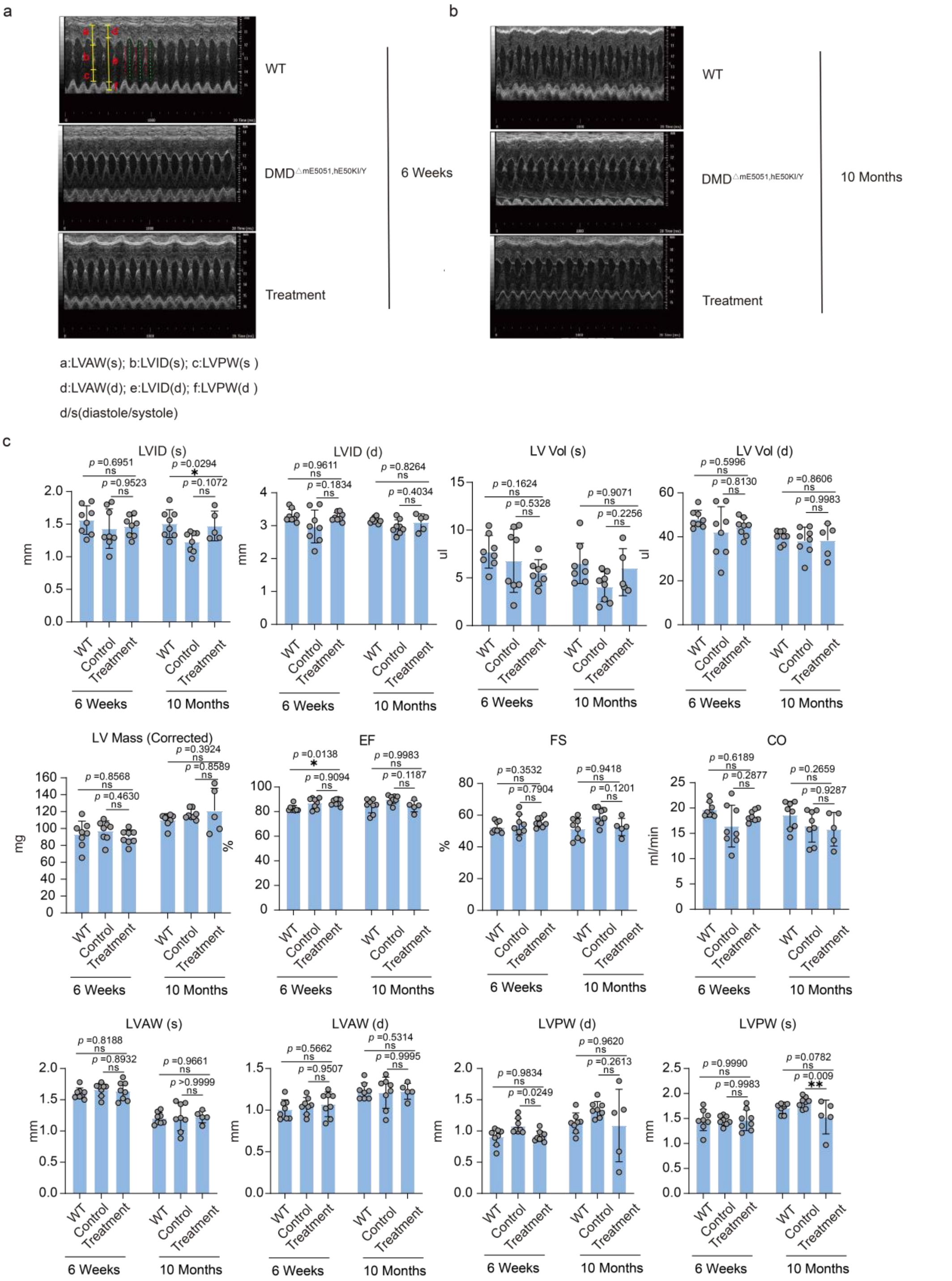

Fig. S16: Echocardiography was used to assess the cardiac function of DMD mice after systemic delivery of ABE2.

a-b, Representative echocardiographic images for DMD$^{\Delta mE5051,KIhE50/Y}$ mice with or without ABE2 administration were monitored for 6 weeks (**a**) and 10 months (**b**). Age-matched WT and DMD mice were included as controls. **c,** Echocardiographic analysis was performed in WT, DMD-mock, and DMD mice

treated with ABE2 after 6 weeks and 10 months injection. LVID;d or LVID;s: Left Ventricular Internal Diameter during diastole or systole; LVPW;d or LVPW;s: Left Ventricular Posterior Wall Thickness during diastole or systole; LVPW;d or LVPW;s: Left Ventricular Posterior Wall Thickness during diastole or systole; LVAW;d or LVAW;s: Left Ventricular Anterior Wall Thickness during diastole or systole; LV Vol;d or LV Vol;s: Left Ventricular Volume during diastole or systole; EF: Ejection Fraction; FS: Fractional Shortening; CO: Cardiac Output; LV Mass (corrected): Left Ventricular Mass corrected for body surface area. Values are shown as mean ± s.d (n=8 independent biological replicates and n=5 independent biological replicates for 10-month post-treatment group). Significance is indicated by asterisk and determined using unpaired two-tailed Student's t test. * $P < 0.05$. ** $P < 0.01$, Ns represents not statistically significant. Source data are provided as a Source Data file.

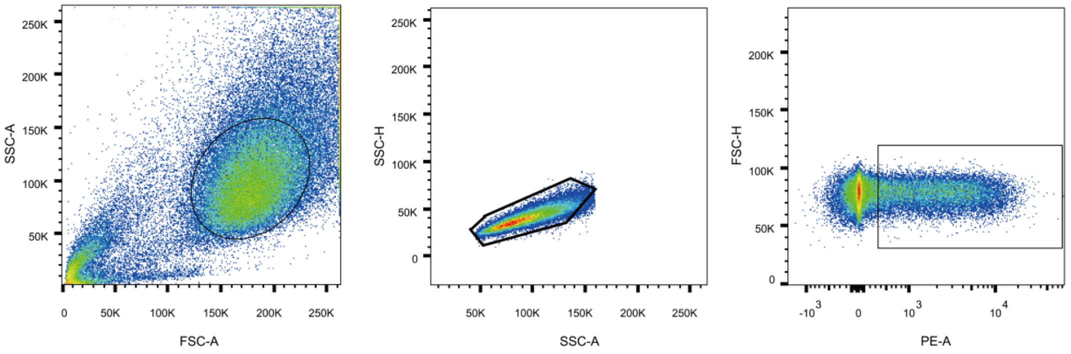

Fig. S17. Flow cytometry gating strategy.

Cell singletons were first gated out via forward scatter (FSC) and side scatter (SSC) parameters. Fluorescent cells were then gated for gene editing analysis. Source data are provided as a Source Data file.

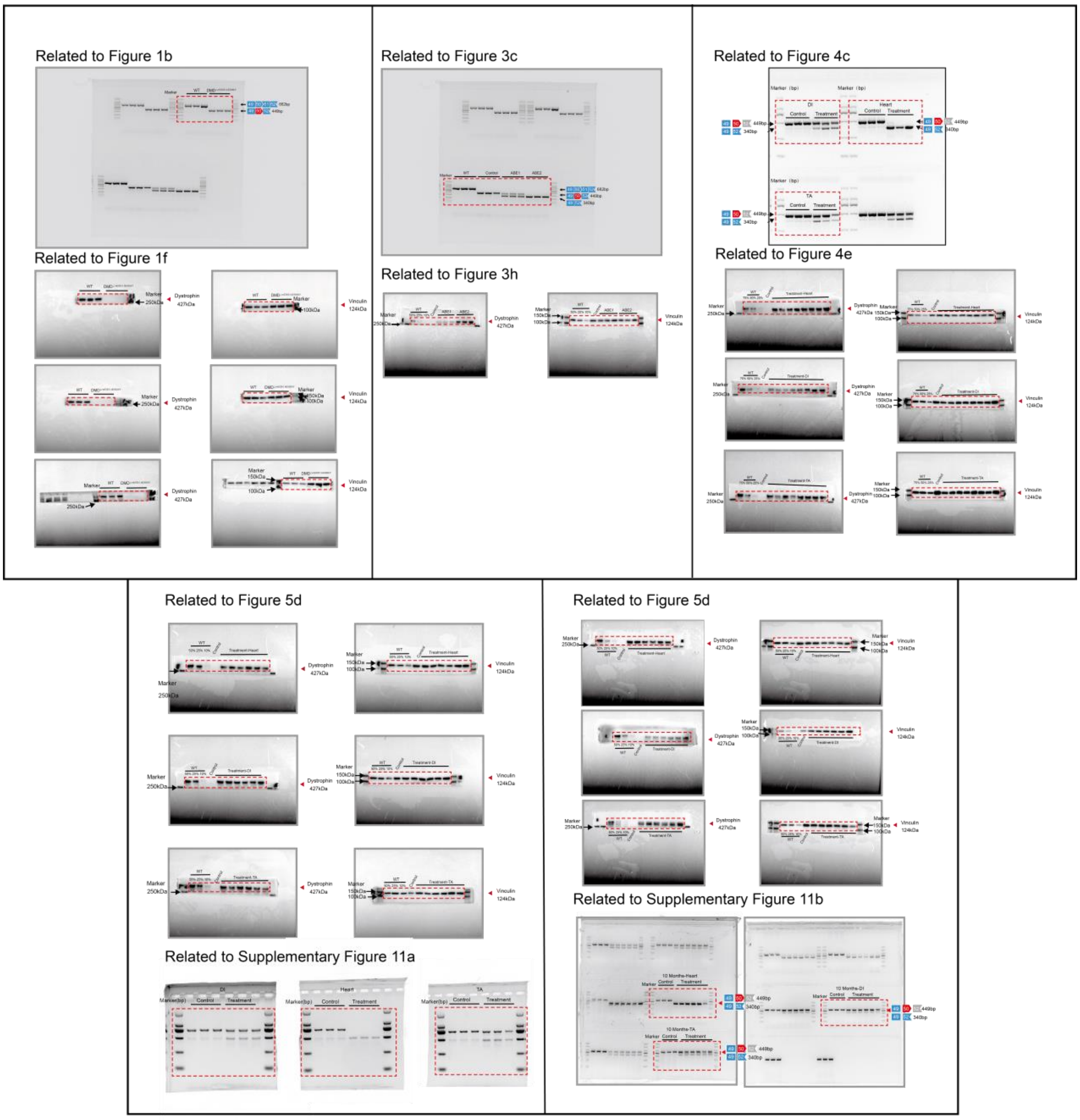

Fig. S18. Uncropped images.

The red rectangles indicate the cropping location.

Table S1: Primer sequence.

Experiment	Primer name	Primer sequence (5'-3')	Product (bp)
Genotyping of DMD$^{\Delta mE5051,KIhE51/Y}$ mice	All-TGF	TTCACTTGCCCTCTTGACC	6791
	All-TGR	GATCAGCAGCCATAAGCTC	
	Intron50-KOF	GCCACATCAGCTCTATCTTCGG	714
	Intron50-KOR	ACAGACAATGGCAATTAAGTCC	
RT-PCR primer flanking exon 50	RNA-E50F	AGATTGAAGTAACAGTTCACGGTA	WT:682<br>Hete:682+449<br>Homo:449<br>Exon skipping:340
	RNA-E50R	TGTTCGGCTTCTTCCTTAGCTT	
Genomic DNA PCR primer flanking exon 50	DNA-E50F	TTGTTCAGGTGCAATACCCACA	975
	DNA-E50R	AATTTAACTGAGCCACTATGCTT	
PCR in different exon	Cell lysis exon2F	TACTGGCCTCAAGTGATCCG	510
	Cell lysis exon2R	CCATATCTTCTGCTGCTTACTCC	
	Cell lysis exon43F	AAGAAAAGAAGTGCAAATACTGA	803
	Cell lysis exon43R	TGTTTATAGCACCTCAATGCC	
	Cell lysis exon44F	AAGAAAATGCCAATAGTCCAAA	792
	Cell lysis exon44R	GGTTCCAACATAAAGCCGAA	
	Cell lysis exon45F	GACAAGAAATCGAATTTGCTCT	759
	Cell lysis exon45R	CCTTTAAGCAATCATGGGT	
	Cell lysis exon46F	TTTAAATTGCCATGTTTGTGTC	337
	Cell lysis exon46R	CTAATGGGCAGAAAACCAAT	
	Cell lysis exon51F	TTATCCCATCTTGTTTTGCCTT	857
	Cell lysis exon51R	ATGGCTACTTTTGTTATTTGCATT	
	Cell lysis exon52F	ATGTCTCCATTTGAGCCTT	683
	Cell lysis exon52R	TGCCAGCCCAGATGACAAC	
	Cell lysis exon53F	ATGGATATTCTGCTGTAGTGCTT	806
	Cell lysis exon53R	CACGCCTGGCTAGTAGTCCC	

	Cell lysis exon55F	GAGCAGCATCAAAGACAAGCA	918
	Cell lysis exon55R	GTTTCTCCTTGACCGAAGCTCT	

Table S2: Target sgRNA sequences.

Experiment	Primer name	Primer sequence (5'-3')
sgRNA for generation $DMD^{\Delta mE5051,KlhE51/Y}$ mice	DMD-T7sgRNA1	CCTGTGATCATGGGTCTAGG
	DMD-T7sgRNA2	ACACACTATTCATTCTACTC
DNA base editing sgRNA in exon 50	sgRNA1	TATACTTACAGGCTCCAAT
	sgRNA2	GTATACTTACAGGCTCCAAT
	sgRNA3	GGTATACTTACAGGCTCCAAT
	sgRNA4	TACTTACAGGCTCCAATAG
	sgRNA5	ATACTTACAGGCTCCAATAG
	sgRNA6	GATACTTACAGGCTCCAATAG
	sgRNA7	GGATACTTACAGGCTCCAATAG
	sgRNA8	TACTTACAGGCTCCAATAGT
	sgRNA9	GTACTTACAGGCTCCAATAGT
	sgRNA10	GGTACTTACAGGCTCCAATAGT
DNA base editing sgRNA in exon 2	sgRNA11	GCATTTTAGATGAAAGAGA
	sgRNA12	CATTTTAGATGAAAGAGA
	sgRNA13	GATTTTAGATGAAAGAGA
	sgRNA14	GCATTTTAGATGAAAGAGAAGA
	sgRNA15	GATTTTAGATGAAAGAGAAGA
	sgRNA16	GTTTTAGATGAAAGAGAAGA
	sgRNA17	GTTTAGATGAAAGAGAAGA
DNA base editing sgRNA in exon 43	sgRNA18	ACCTACCCTTGTCGGTCCT
	sgRNA19	TACCTACCCTTGTCGGTCCT
	sgRNA20	GTACCTACCCTTGTCGGTCCT
	sgRNA21	GGTACCTACCCTTGTCGGTCCT

DNA base editing sgRNA in exon 44	sgRNA22	GTACCTGCAGGCGATTTGAC
	sgRNA23	GCCTGCAGGCGATTTGAC
	sgRNA24	GCTGCAGGCGATTTGACAGATC
	sgRNA25	GTGCAGGCGATTTGACAGATC
	sgRNA26	TGCAGGCGATTTGACAGATC
	sgRNA27	CTTACCTTAAGATACCATT
	sgRNA28	ACTTACCTTAAGATACCATT
	sgRNA29	GACTTACCTTAAGATACCATT
	sgRNA30	GGACTTACCTTAAGATACCATT
DNA base editing sgRNA in exon 45	sgRNA31	GATCTTACAGGAACTCCAGGA
	sgRNA32	GTCTTACAGGAACTCCAGGA
	sgRNA33	GCTTACAGGAACTCCAGGA
	sgRNA34	GTTACAGGAACTCCAGGA
	sgRNA35	GTCTTACAGGAACTCCAGGAT
	sgRNA36	GCTTACAGGAACTCCAGGAT
	sgRNA37	GTTACAGGAACTCCAGGAT
	sgRNA38	GTACAGGAACTCCAGGAT
DNA base editing sgRNA in exon 46	sgRNA39	GAGCAAGTCAAGGTAATTT
	sgRNA40	TGAGCAAGTCAAGGTAATTT
	sgRNA41	GTGAGCAAGTCAAGGTAATTT
	sgRNA42	GGTGAGCAAGTCAAGGTAATTT
DNA base editing sgRNA in exon 51	sgRNA43	TTTTCTCATACCTTCTGCT
	sgRNA44	TCTCATACCTTCTGCTTGA
	sgRNA45	TTCTCATACCTTCTGCTTGA
	sgRNA46	GTTCTCATACCTTCTGCTTGA
	sgRNA47	GGTTCTCATACCTTCTGCTTGA
DNA base editing sgRNA in exon 52	sgRNA48	AACTTACTTCGATCCGTAA
	sgRNA49	AAACTTACTTCGATCCGTAA
	sgRNA50	GAAACTTACTTCGATCCGTAA

	sgRNA51	GGAAACTTACTTCGATCCGTAA
DNA base editing sgRNA in exon 53	sgRNA52	TGATACTAACCTTGGTTTC
	sgRNA53	TTGATACTAACCTTGGTTTC
	sgRNA54	GTTGATACTAACCTTGGTTTC
	sgRNA55	ATACTAACCTTGGTTTCTG
DNA base editing sgRNA in exon 55	sgRNA56	GTCCTTTGCAGGGTGAGTGAG
	sgRNA57	GCCTTTGCAGGGTGAGTGAG
	sgRNA58	GCTTTGCAGGGTGAGTGAG
	sgRNA59	GTTTGCAGGGTGAGTGAG
	sgRNA60	GCTTTGCAGGGTGAGTGAGCG
	sgRNA61	GTTTGCAGGGTGAGTGAGCG
	sgRNA62	GTTGCAGGGTGAGTGAGCG
	sgRNA63	GTGCAGGGTGAGTGAGCG
	sgRNA64	GTTTGCAGGGTGAGTGAGCG
	sgRNA65	GTTGCAGGGTGAGTGAGCG
	sgRNA66	GTGCAGGGTGAGTGAGCG
	sgRNA67	GTTGCAGGGTGAGTGAGCGAG
	sgRNA68	GTGCAGGGTGAGTGAGCGAG
	sgRNA69	TGCAGGGTGAGTGAGCGAG
	sgRNA70	TTGCAGGGTGAGTGAGCGAGA
	sgRNA71	GTGCAGGGTGAGTGAGCGAGA

Supplementary Note 1. Sequences of ABE1 and ABE2.

hU6-Spacer-SpCas9 sgRNA scaffiod-Spc5-12-BPNLS-TadA8e-Linker-Cas9n (D10A)-N-Rma-N-W3SL

gagggcctatttcccatgattccttcatatttgcatatacgatacaaggctgttagagagataattggaattaatttgactgtaaacacaaagatattagtacaaaatacgtgacgtagaaagtaataatttcttgggtagtttgcagttttaaaattatgttttaaaatggactatcatatgcttaccgtaacttgaaagtatttcgatttcttggctttatatatcttGTGGAAAGGACGAAACACCGATACTTACAGGCTCCAATAGgttttagagctaGAAAtagcaagttaaaataaggctagtccgttatcaacttgaaaaagtggcaccgagtcggtgcTTTTTTgagggcctatttcccatgattccttcatatttgcatatacgatacaaggctgttagagagataattggaattaatttgactgtaaacacaaagatattagtacaaaatacgtgacgtagaaagtaataatttcttgggtagtttgcagttttaaaattatgttttaaaatggactatcatatgcttaccgtaacttgaaagtatttcgatttcttggctttatatatcttGTGGAAAGGACGAAACACCGATACTTACAGGCTCCAATAGgttttagagctaGAAAtagcaagttaaaataaggctagtccgttatcaacttgaaaaagtggcaccgagtcggtgcTttttttttttcaccgcggtggcggccgtccgccctcggcaccatcctcacgacacccaaatatggcgacgggtgaggaatggtggggagttatttttagagcggtgaggaaggtgggcaggcagcaggtgttggcgctctaaaaataactcccgggagttatttttagagcggaggaatggtggacacccaaatatggcgaccggttcctcaaccggtcgccatatttgggtgtccgccctcggccggggccgcattcctgggggccgggcggtgctcccgcccgcctcgataaaaggctccggggccggcggcggcccacgagcaccggtgccaccaccggtgccaccatgaaacggacagccgacggaagcgagttcgagtcaccaaagaagaagcggaaagtctctgaggtggagttttcccacgagtactggatgagacatgccctgaccctggccaagagggcacgcgatgagagggaggtgcctgtgggagccgtgctggtgctgaacaatagagtgatcggcgagggctggaacagagccatcggcctgcacgacccaacagcccatgccgaaattatggccctgagacagggcggcctggtcatgcagaactacagactgattgacgccaccctgtacgtgacattcgagccttgcgtgatgtgcgccggcgccatgatccactctaggatcggccgcgtggtgtttggcgtgaggaacTCAaaaAGAggcgccgcaggCTCCCTGATGAACGTGCTGAACTACCCCGGCATGAATCACCGCGTCGAAATTACCGAGGGAATCCTGGCAGATGAATGTGCCGCCCTGCTGTGCGATTTCTATCGGATGCCTAGACAGGTGTTCAATGCTCAGAAGAaggcccagagctccATCAACtccggaggatctagcggaggctcctctggctctgagacacctggcacaagcgagagcgcaacacctgaaagcagcgggggcagcagcgggggtcagacaagaagtacagcatcggcctggccatcggcaccaactctgtgggctgggccgtgatcaccgac

gagtacaaggtgcccagcaagaaattcaaggtgctgggcaacaccgaccggcacagcatcaagaaga
acctgatcggagccctgctgttcgacagcggcgaaacagccgaggccacccggctgaagagaaccgcc
agaagaagatacaccagacggaagaaccggatctgctatctgcaagagatcttcagcaacgagatggc
caaggtggacgacagcttcttccacagactggaagagtccttcctggtggaagaggataagaagcacga
gcggcaccccatcttcggcaacatcgtggacgaggtggcctaccacgagaagtaccccaccatctacca
cctgagaaagaaactggtggacagcaccgacaaggccgacctgcggctgatctatctggccctggccca
catgatcaagttccggggccacttcctgatcgagggcgacctgaaccccgacaacagcgacgtggacaa
gctgttcatccagctggtgcagacctacaaccagctgttcgaggaaaaccccatcaacgccagcggcgtg
gacgccaaggccatcctgtctgccagactgagcaagagcagacggctggaaaatctgatcgcccagctg
cccggcgagaagaagaatggcctgttcggaaacctgattgccctgagcctgggcctgacccccaacttca
agagcaacttcgacctggccgaggatgccaaactgcagctgagcaaggacacctacgacgacgacctg
gacaacctgctggcccagatcggcgaccagtacgccgacctgtttctggccgccaagaacctgtccgacg
ccatcctgctgagcgacatcctgagagtgaacaccgagatcaccaaggcccccctgagcgcctctatgat
caagagatacgacgagcaccaccaggacctgaccctgctgaaagctctcgtgcggcagcagctgcctga
gaagtacaaagagattttcttcgaccagagcaagaacggctacgccggctacattgacggcggagccag
ccaggaagagttctacaagttcatcaagcccatcctggaaaagatggacggcaccgaggaactgctcgtg
aagctgaacagagaggacctgctgcggaagcagcggaccttcgacaacggcagcatcccccaccaga
tccacctgggagagctgcacgccattctgcggcggcaggaagatttttacccattcctgaaggacaaccgg
gaaaagatcgagaagatcctgaccttccgcatcccctactacgtgggccctctggccaggggaaacagc
agattcgcctggatgaccagaaagagcgaggaaaccatcaccccctggaacttcgaggaagtggtgga
caagggcgcttccgcccagagcttcatcgagcggatgaccaacttcgataagaacctgcccaacgagaa
ggtgctgcccaagcacagcctgctgtacgagtacttcaccgtgtataacgagctgaccaaagtgaaatacg
tgaccgagggaatgagaaagcccgccttcctgagcggcgagcagaaaaaggccatcgtggacctgctgt
tcaagaccaaccggaaagtgaccgtgaagcagctgaaagaggactacttcaagaaaatcgagTGTC
TGGCTGGCGATACTCTCATTACCCTGGCCGATGGACGACGAGTGCCTATT
AGAGAACTGGTGTCACAGCAGAATTTTTCCGTGTGGGCTCTGAATCCTCA
GACTTACCGCCTGGAGAGGGCTAGAGTGAGTAGAGCTTTCTGTACCGGC
ATCAAACCTGTGTACCGCCTCACCACTAGACTGGGGAGATCCATTAGGGC
CACTGCCAACCACCGATTTCTCACACCTCAGGGCTGGAAACGAGTCGAT
GAACTCCAGCCTGGAGATTACCTGGCTCTGCCTAGGAGAATCCCTACTGC

CTCCTGAgaattcCGCTCGAGATAATCAACCTCTGGATTACAAAATTTGTGAAAGATTGACTGGTATTCTTAACTATGTTGCTCCTTTTACGCTATGTGGATACGCTGCTTTAATGCCTTTGTATCATGCTATTGCTTCCCGTATGGCTTTCATTTTCTCCTCCTTGTATAAATCCTGGTTAGTTCTTGCCACGGCGGAACTCATCGCCGCCTGCCTTGCCCGCTGCTGGACAGGGGCTCGGCTGTTGGGCACTGACAATTCCGTGGTGTTTATTTGTGAAATTTGTGATGCTATTGCTTTATTTGTAACCATCTAGCTTTATTTGTGAAATTTGTGATGCTATTGCTTTATTTGTAACCATTATAAGCTGCAATAAACAAGTTAACAACAACAATTGCATTCATTTTATGTTTCAGGTTCAGGGGGAGATGTGGGAGGTTTTTTAAA

Spc5-12-Rma-C-Cas9n(D10A)-C-BPNLS-Linker-W3SL

caccgcggtggcggccgtccgccctcggcaccatcctcacgacacccaaatatggcgacgggtgaggaatggtggggagttatttttagagcggtgaggaaggtgggcaggcagcaggtgttggcgctctaaaaataactcccgggagttatttttagagcggaggaatggtggacacccaaatatggcgaccggttcctcaaccggtcgccatatttgggtgtccgccctcggccggggccgcattcctgggggccgggcggtgctcccgcccgcctcgataaaaggctccggggccggcggcggcccacgagcaagcttgccaccatgatggcggcggcgtgcccggaactgcgtcagctggcgcagagcgatgtgtattgggatccgattgtgagcattgaaccggatggcgtggaagaagtgtttgatctgaccgtgccgggcccgcataactttgtggcgaacgatattattgcgcataactgcttcgactccgtggaaatctccggcgtggaagatcggttcaacgcctccctgggcacataccacgatctgctgaaaattatcaaggacaaggacttcctggacaatgaggaaaacgaggacattctggaagatatcgtgctgaccctgacactgtttgaggacagagagatgatcgaggaacggctgaaaacctatgcccacctgttcgacgacaaagtgatgaagcagctgaagcggcggagatacaccggctggggcaggctgagccggaagctgatcaacggcatccgggacaagcagtccggcaagacaatcctggatttcctgaagtccgacggcttcgccaacagaaacttcatgcagctgatccacgacgacagcctgacctttaaagaggacatccagaaagcccaggtgtccggccagggcgatagcctgcacgagcacattgccaatctggccggcagccccgccattaagaagggcatcctgcagacagtgaaggtggtggacgagctcgtgaaagtgatgggccggcacaagcccgagaacatcgtgatcgaaatggccagagagaaccagaccacccagaagggacagaagaacagccgcgagagaatgaagcggatcgaagagggcatcaaagagctgggcagccagatcctgaaagaacaccccgtggaaaacacccagctgcagaacgagaagctgtacctgtactacctgcagaatgggcgggatatgtacgtggaccaggaactggacatcaaccggctgtccgactacgatgtggaccatatcgtgcctcagagctttctgaaggacgactccat

cgacaacaaggtgctgaccagaagcgacaagaaccggggcaagagcgacaacgtgccctccgaaga
ggtcgtgaagaagatgaagaactactggcggcagctgctgaacgccaagctgattacccagagaaagtt
cgacaatctgaccaaggccgagagaggcggcctgagcgaactggataaggccggcttcatcaagagac
agctggtggaaacccggcagatcacaaagcacgtggcacagatcctggactcccggatgaacactaagt
acgacgagaatgacaagctgatccgggaagtgaaagtgatcaccctgaagtccaagctggtgtccgattt
ccggaaggatttccagttttacaaagtgcgcgagatcaacaactaccaccacgcccacgacgcctacctg
aacgccgtcgtgggaaccgccctgatcaaaaagtaccctaagctggaaagcgagttcgtgtacggcgact
acaaggtgtacgacgtgcggaagatgatcgccaagagcgagcaggaaatcggcaaggctaccgccaa
gtacttcttctacagcaacatcatgaactttttcaagaccgagattaccctggccaacggcgagatccggaa
gcggcctctgatcgagacaaacggcgaaaccggggagatcgtgtgggataagggccgggattttgccac
cgtgcggaaagtgctgagcatgccccaagtgaatatcgtgaaaaagaccgaggtgcagacaggcggctt
cagcaaagagtctatcctgcccaagaggaacagcgataagctgatcgccagaaagaaggactgggac
cctaagaagtacggcggcttcCTTTGGcccaccgtggcctattctgtgctggtggtggccaaagtggaa
aagggcaagtccaagaaactgaagagtgtgaaagagctgctggggatcaccatcatggaaagaagca
gcttcgagaagaatcccatcgactttctggaagccaagggctacaaagaagtgaaaaaggacctgatcat
caagctgcctaagtactccctgttcgagctggaaaacggccggaagagaatgctggcctctgccAAGC
AActgcagaagggaaacgaactggccctgccctccaaatatgtgaacttcctgtacctggccagccactat
gagaagctgaagggctcccccgaggataatgagcagaaacagctgtttgtggaacagcacaagcacta
cctggacgagatcatcgagcagatcagcgagttctccaagagagtgatcctggccgacgctaatctggac
aaagtgctgtccgcctacaacaagcaccgggataagcccatcagagagcaggccgagaatatcatcca
cctgtttaccctgaccaatctgggagcccctgccgccttcaagtactttgacaccaccatcgaccggaagC
AAtacAGAagcaccaaagaggtgctggacgccaccctgatccaccagagcatcaccggcctgtacga
gacacggatcgacctgtctcagctgggaggtgactctggcggctcaaaaagaaccgccgacggcagcg
aattcgagcccaagaagaagaggaaagtctaagaattcCGCTCGAGATAATCAACCTCTG
GATTACAAAATTTGTGAAAGATTGACTGGTATTCTTAACTATGTTGCTCCTT
TTACGCTATGTGGATACGCTGCTTTAATGCCTTTGTATCATGCTATTGCTTC
CCGTATGGCTTTCATTTTCTCCTCCTTGTATAAATCCTGGTTAGTTCTTGCC
ACGGCGGAACTCATCGCCGCCTGCCTTGCCCGCTGCTGGACAGGGGCT
CGGCTGTTGGGCACTGACAATTCCGTGGTGTTTATTTGTGAAATTTGTGAT
GCTATTGCTTTATTTGTAACCATCTAGCTTTATTTGTGAAATTTGTGATGCTA

TTGCTTTATTTGTAACCATTATAAGCTGCAATAAACAAGTTAACAACAACAATTGCATTCATTTTATGTTTCAGGTTCAGGGGGAGATGTGGGAGGTTTTTTAAA

hU6-Spacer-SpCas9 sgRNA scaffiod-Spc5-12-Rma-C-Cas9n(D10A)-C-BPNLS-Linker-W3SL

gagggcctatttcccatgattccttcatatttgcatatacgatacaaggctgttagagagataattggaattaattgactgtaaacacaaagatattagtacaaaatacgtgacgtagaaagtaataatttcttgggtagtttgcagttttaaaattatgttttaaaatggactatcatatgcttaccgtaacttgaaagtatttcgatttcttggctttatatatcttGTGGAAAGGACGAAACACCGATACTTACAGGCTCCAATAGgttttagagctaGAAAtagcaagttaaaataaggctagtccgttatcaacttgaaaaagtggcaccgagtcggtgcTTTTTTcaccgcggtggcggccgtccgccctcggcaccatcctcacgacacccaaatatggcgacgggtgaggaatggtggggagttatttttagagcggtgaggaaggtgggcaggcagcaggtgttggcgctctaaaaataactcccgggagttatttttagagcggaggaatggtggacacccaaatatggcgaccggttcctcaaccggtcgccatatttgggtgtccgccctcggccggggccgcattcctgggggccgggcggtgctcccgcccgcctcgataaaaggctccggggccggcggcggcccacgagcaagcttgccaccatgatggcggcggcgtgcccggaactgcgtcagctggcgcagagcgatgtgtattgggatccgattgtgagcattgaaccggatggcgtggaagaagtgtttgatctgaccgtgccgggcccgcataactttgtggcgaacgatattattgcgcataactgcttcgactccgtggaaatctccggcgtggaagatcggttcaacgcctccctgggcacataccacgatctgctgaaaattatcaaggacaaggacttcctggacaatgaggaaaacgaggacattctggaagatatcgtgctgaccctgacactgtttgaggacagagagatgatcgaggaacggctgaaaacctatgcccacctgttcgacgacaaagtgatgaagcagctgaagcggcggagatacaccggctggggcaggctgagccggaagctgatcaacggcatccgggacaagcagtccggcaagacaatcctggatttcctgaagtccgacggcttcgccaacagaaacttcatgcagctgatccacgacgacagcctgacctttaaagaggacatccagaaagcccaggtgtccggccagggcgatagcctgcacgagcacattgccaatctggccggcagccccgccattaagaagggcatcctgcagacagtgaaggtggtggacgagctcgtgaaagtgatgggccggcacaagcccgagaacatcgtgatcgaaatggccagagagaaccagaccacccagaagggacagaagaacagccgcgagagaatgaagcggatcgaagagggcatcaaagagctgggcagccagatcctgaaagaacaccccgtggaaaacacccagctgcagaacgagaagctgtacctgtactacctgcagaatggcgggatatgtacgtggaccaggaactggacatcaaccggctgtccgactacgatgtggaccatatcgtgcctcagagctttctgaaggacgactccatcg

acaacaaggtgctgaccagaagcgacaagaaccggggcaagagcgacaacgtgccctccgaagaggtcgtgaagaagatgaagaactactggcggcagctgctgaacgccaagctgattacccagagaaagttcgacaatctgaccaaggccgagagaggcggcctgagcgaactggataaggccggcttcatcaagagacagctggtggaaacccggcagatcacaaagcacgtggcacagatcctggactcccggatgaacactaagtacgacgagaatgacaagctgatccgggaagtgaaagtgatcaccctgaagtccaagctggtgtccgatttccggaaggatttccagttttacaaagtgcgcgagatcaacaactaccaccacgcccacgacgcctacctgaacgccgtcgtgggaaccgccctgatcaaaaagtaccctaagctggaaagcgagttcgtgtacggcgactacaaggtgtacgacgtgcggaagatgatcgccaagagcgagcaggaaatcggcaaggctaccgccaagtacttcttctacagcaacatcatgaactttttcaagaccgagattaccctggccaacggcgagatccggaagcggcctctgatcgagacaaacggcgaaaccggggagatcgtgtgggataagggccgggattttgccaccgtgcggaaagtgctgagcatgccccaagtgaatatcgtgaaaaagaccgaggtgcagacaggcggcttcagcaaagagtctatcctgcccaagaggaacagcgataagctgatcgccagaaagaaggactgggaccctaagaagtacggcggcttcCTTTGGcccaccgtggcctattctgtgctggtggtggccaaagtggaaaagggcaagtccaagaaactgaagagtgtgaaagagctgctggggatcaccatcatggaaagaagcagcttcgagaagaatcccatcgactttctggaagccaagggctacaaagaagtgaaaaaggacctgatcatcaagctgcctaagtactccctgttcgagctggaaaacggccggaagagaatgctggcctctgccAAGCAActgcagaagggaaacgaactggccctgccctccaaatatgtgaacttcctgtacctggccagccactatgagaagctgaagggctcccccgaggataatgagcagaaacagctgtttgtggaacagcacaagcactacctggacgagatcatcgagcagatcagcgagttctccaagagagtgatcctggccgacgctaatctggacaaagtgctgtccgcctacaacaagcaccgggataagcccatcagagagcaggccgagaatatcatccacctgtttaccctgaccaatctgggagcccctgccgccttcaagtactttgacaccaccatcgaccggaagCAAtacAGAagcaccaaagaggtgctggacgccaccctgatccaccagagcatcaccggcctgtacgagacacggatcgacctgtctcagctgggaggtgactctggcggctcaaaaagaaccgccgacggcagcgaattcgagcccaagaagaagaggaaagtctaagaattcCGCTCGAGATAATCAACCTCTGGATTACAAAATTTGTGAAAGATTGACTGGTATTCTTAACTATGTTGCTCCTTTTACGCTATGTGGATACGCTGCTTTAATGCCTTTGTATCATGCTATTGCTTCCCGTATGGCTTTCATTTTCTCCTCCTTGTATAAATCCTGGTTAGTTCTTGCCACGGCGGAACTCATCGCCGCCTGCCTTGCCCGCTGCTGGACAGGGGCTCGGCTGTTGGGCACTGACAATTCCGTGGTGTTTATTTGTGAAATTTGTGATGCTATTGCTTTATTTGTAACCATCTAGCTTTATTTGTGAAATTTGTGATGCTA

TTGCTTTATTTGTAACCATTATAAGCTGCAATAAACAAGTTAACAACAACAA
TTGCATTCATTTTATGTTTCAGGTTCAGGGGGAGATGTGGGAGGTTTTTTA
AA
