# Supplementary material for: Adenine base editing-mediated exon skipping restores dystrophin in humanized Duchenne mouse model
Source: Nat Commun. 2024 Jul 15;15:5927. doi: 10.1038/s41467-024-50340-x (PMC11251194; doi:10.1038/s41467-024-50340-x)
Supplement: Supplementary file 2 — Reporting Summary [file 41467_2024_50340_MOESM2_ESM.pdf]

nature portfolio

Corresponding author(s): Guoling Li,Chunlong Xu, Hui Yang, Shiwen Wu, Ning Wang

NCOMMS-23-19849C

Last updated by author(s): Jun 18, 2024

Reporting Summary

Nature Portfolio wishes to improve the reproducibility of the work that we publish. This form provides structure for consistency and transparency in reporting. For further information on Nature Portfolio policies, see our Editorial Policies and the Editorial Policy Checklist.

Statistics

For all statistical analyses, confirm that the following items are present in the figure legend, table legend, main text, or Methods section.

n/a	Confirmed	
☐	☒	The exact sample size (*n*) for each experimental group/condition, given as a discrete number and unit of measurement
☐	☒	A statement on whether measurements were taken from distinct samples or whether the same sample was measured repeatedly
☐	☒	The statistical test(s) used AND whether they are one- or two-sided<br>*Only common tests should be described solely by name; describe more complex techniques in the Methods section.*
☒	☐	A description of all covariates tested
☒	☐	A description of any assumptions or corrections, such as tests of normality and adjustment for multiple comparisons
☐	☒	A full description of the statistical parameters including central tendency (e.g. means) or other basic estimates (e.g. regression coefficient) AND variation (e.g. standard deviation) or associated estimates of uncertainty (e.g. confidence intervals)
☐	☒	For null hypothesis testing, the test statistic (e.g. *F*, *t*, *r*) with confidence intervals, effect sizes, degrees of freedom and *P* value noted<br>*Give P values as exact values whenever suitable.*
☒	☐	For Bayesian analysis, information on the choice of priors and Markov chain Monte Carlo settings
☒	☐	For hierarchical and complex designs, identification of the appropriate level for tests and full reporting of outcomes
☒	☐	Estimates of effect sizes (e.g. Cohen's *d*, Pearson's *r*), indicating how they were calculated

Our web collection on statistics for biologists contains articles on many of the points above.

Software and code

Policy information about availability of computer code

Data collection	No public data was collected in the study.
Data analysis	Flow cytometry data was analyzed by FlowJo X(v10.0.7). Statistics analysis were performed with Prism 9.

For manuscripts utilizing custom algorithms or software that are central to the research but not yet described in published literature, software must be made available to editors and reviewers. We strongly encourage code deposition in a community repository (e.g. GitHub). See the Nature Portfolio guidelines for submitting code & software for further information.

Data

Policy information about availability of data

All manuscripts must include a data availability statement. This statement should provide the following information, where applicable:

- Accession codes, unique identifiers, or web links for publicly available datasets
- A description of any restrictions on data availability
- For clinical datasets or third party data, please ensure that the statement adheres to our policy

All the sequencing data have been deposited in the NCBI SRA under project accession number PRJNA1105327. Source data are provided with this paper.

Research involving human participants, their data, or biological material

Policy information about studies with human participants or human data. See also policy information about sex, gender (identity/presentation), and sexual orientation and race, ethnicity and racism.

Reporting on sex and gender	N/A
Reporting on race, ethnicity, or other socially relevant groupings	N/A
Population characteristics	N/A
Recruitment	N/A
Ethics oversight	N/A

Note that full information on the approval of the study protocol must also be provided in the manuscript.

Field-specific reporting

Please select the one below that is the best fit for your research. If you are not sure, read the appropriate sections before making your selection.

[x] Life sciences [ ] Behavioural & social sciences [ ] Ecological, evolutionary & environmental sciences

For a reference copy of the document with all sections, see nature.com/documents/nr-reporting-summary-flat.pdf

Life sciences study design

All studies must disclose on these points even when the disclosure is negative.

Sample size	No statistical analysis were performed to predetermine sample size in this study. But sample sizes in this study are in accordance to those generaly used in genome editing experiments. All cellular experiments were conducted with two to three biologically independent replicates. For all animal experiments, between three and nine mice were used.
Data exclusions	No data was excluded.
Replication	All main text figure experiments were repeated at least three times and all attempts at replication were successful.
Randomization	Human cells were grown at identical condition, after seeding cells into 24-wells plates, we randomly selected cells for test group and control group. DMD mice used for gene editing therapy were allocated to control or AAV9 treated group randomly.
Blinding	No blinding was applied, due to no subjective assessments were required.

Reporting for specific materials, systems and methods

We require information from authors about some types of materials, experimental systems and methods used in many studies. Here, indicate whether each material, system or method listed is relevant to your study. If you are not sure if a list item applies to your research, read the appropriate section before selecting a response.

Materials & experimental systems

n/a	Involved in the study	
[ ]	[x]	Antibodies
[ ]	[x]	Eukaryotic cell lines
[x]	[ ]	Palaeontology and archaeology
[ ]	[x]	Animals and other organisms
[x]	[ ]	Clinical data
[x]	[ ]	Dual use research of concern
[x]	[ ]	Plants

Methods

n/a	Involved in the study	
[x]	[ ]	ChIP-seq
[ ]	[x]	Flow cytometry
[x]	[ ]	MRI-based neuroimaging

Antibodies

Antibodies used	Antibodies for western blot: primary antibodies against dystrophin (1:1000 dilution, Sigma, D8168) and vinculin (1:1000 dilution, CST, 13901S); secondary antibody (1:1000 dilution, Beyotime, A0216) ;Antibodies for immunofluorescence: primary antibodies against dystrophin (1:100 dilution, Abcam, ab15277), spectrin (1:500 dilution, Millipore, MAB1622), PAX7 (1:500dilution, DSHB, 042349) and

	PAX3 (1:500 dilution, Beyotime, AF7686); secondary antibodies (Alexa Fluor 488 AffiniPure donkey anti-rabbit IgG (1:1000 dilution, Jackson ImmunoResearch labs, 711-545-152) or Alexa Fluor 647 AffiniPure donkey anti-mouse IgG (1:1000 dilution, Jackson ImmunoResearch labs, 715-605-151))
Validation	Validation information is available at the following link: https://www.sigmaaldrich.com/HK/zh/product/sigma/d8168; https://www.cellsignal.com/products/primary-antibodies/vinculin-e1e9v-xp-rabbit-mab/13901; https://www.abcam.com/products/primaryantibodies/dystrophin-antibody-ab15277.html; https://www.merckmillipore.com/HK/en/product/msds/MM_NF-MAB1622; https://dshb.biology.uiowa.edu/PAX7; https://www.beyotime.com/product/AF7686.htm;ReferrerURL=https%3A%2F%2Fwww.bing.com%2F

Eukaryotic cell lines

Policy information about cell lines and Sex and Gender in Research

Cell line source(s)	HEK293T cells were purchased from Stem Cell Bank, Chinese Academy of Sciences.
Authentication	HEK293T cells were validated by supplier.
Mycoplasma contamination	All cell lines tested negative for mycoplasma contamination by PCR.
Commonly misidentified lines (See ICLAC register)	None misidentified lines were used.

Animals and other research organisms

Policy information about studies involving animals; ARRIVE guidelines recommended for reporting animal research, and Sex and Gender in Research

Laboratory animals	C57 BL/6J, mouse DMD exon50,exon51 replace with human DMD exon50 flanking intron 200bp, neonatal, 2weeks, 3weeks, 4weeks, 8weeks, 24weeks and 10months old. Mice were housed in a barrier facility with a 12-hour light/dark cycle and 18-23°C with 40-60% humidity. Diet and water were be accessible at all times. All of these were maintained in accordance with the Instructive Notions with Respect to Caring for Laboratory Animals issued by the Ministry of Science and Technology of China.<br>Wild-type C57BL/6 mice were used in this study.<br>DMD mice were generated in the C57BL/6J background using the CRISPR-Cas9 system. In brief, two sgRNAs targeted mouse DMD intron50 and intron52 were designed, and then T7 promoter sequence was added to the sgRNA template. After PCR product purified directly with Omega gel extraction kit, templates were used for in vitro transcription using the MEGAshortscript T7 Kit. sgRNAs were purified by MEGAclear Kit and eluted with nuclease-free water. For cytoplasmic injection, spCas9 mRNA (100 ng/!l), sgRNA (100 ng/!l) and HMEJ donor (100 ng/!l) were mixed, and then injected into the fertilized eggs using a FemtoJet microinjector with constant flow settings. The injected zygotes were cultured in KSOM medium with 12 hours, and surgically transferred to the oviduct of recipient mice 24 hours after estrus was observed.<br>After AAV9 intramuscular injection 6weeks,mice were anesthetized, euthanized and TA (tibialis anterior) muscle was collection. DMD mice were injected with viral particles systemically via tail vein (2 weeks old mice) and intraperitoneal approach (P4 mice).
Wild animals	No wild animals are involved in the study.
Reporting on sex	Duchenne muscular dystrophy (DMD) is the most common sex linked lethal disease in man, thus male mice were selected for this study.
Field-collected samples	This study did not involve samples collected from the field.
Ethics oversight	All animal experiments were performed and approved by the Animal Care and Use Committee of Huidagene Therapeutics Co., Ltd, Shanghai, China.

Note that full information on the approval of the study protocol must also be provided in the manuscript.

Plants

Seed stocks	N/A
Novel plant genotypes	N/A
Authentication	N/A

Flow Cytometry

Plots

Confirm that:

- [x] The axis labels state the marker and fluorochrome used (e.g. CD4-FITC).
- [x] The axis scales are clearly visible. Include numbers along axes only for bottom left plot of group (a 'group' is an analysis of identical markers).
- [x] All plots are contour plots with outliers or pseudocolor plots.
- [x] A numerical value for number of cells or percentage (with statistics) is provided.

Methodology

Sample preparation	Post-transfected cells were trypsinized, resuspended with cell culture medium, and analyzed by flow cytometry.
Instrument	BD FACSAria III was used for cell sorting
Software	FlowJo X(v10.0.7)
Cell population abundance	N/A
Gating strategy	Gating strategy for reporter assay was provided in supplementary figures.

- [x] Tick this box to confirm that a figure exemplifying the gating strategy is provided in the Supplementary Information.
